# Morphological and Functional Inner and Outer Retinal Layer Abnormalities in Eyes with Permanent Temporal Hemianopia from Chiasmal Compression

**DOI:** 10.3389/fneur.2017.00619

**Published:** 2017-12-04

**Authors:** Rafael B. de Araújo, Maria K. Oyamada, Leandro C. Zacharias, Leonardo P. Cunha, Rony C. Preti, Mário L. R. Monteiro

**Affiliations:** ^1^Laboratory of Investigation in Ophthalmology (LIM 33), Division of Ophthalmology, University of São Paulo Medical School, São Paulo, Brazil; ^2^Department of Ophtalmology, School of Medicine, Federal University of Juiz de Fora, Juiz de Fora, Brazil

**Keywords:** optical coherence tomography, oscillatory potentials, multifocal electroretinography, standard automated perimetry, chiasmal compression, temporal hemianopia

## Abstract

**Purpose:**

The aims of this study are to compare optical coherence tomography (OCT)-measured macular retinal layers in eyes with permanent temporal hemianopia from chiasmal compression and control eyes; to compare regular and slow-flash multifocal electroretinography (mfERG) in patients and controls; and to assess the correlation between OCT, mfERG, and central visual field (SAP) data.

**Methods:**

Forty-three eyes of 30 patients with permanent temporal hemianopia due to pituitary tumors who were previously submitted to chiasm decompression and 37 healthy eyes of 19 controls were submitted to macular spectral domain OCT, mfERG, and 10-2 SAP testing. After segmentation, the thickness of the macular retinal nerve fiber layer (RNFL), ganglion cell layer (GCL), inner plexiform layer (IPL), inner nuclear layer (INL), outer plexiform layer (OPL), outer nuclear layer, and photoreceptor layer (PRL) was measured. Amplitudes and oscillatory potentials (OPs) were measured on regular and slow-flash mfERG, respectively, and expressed as the mean values per quadrant and hemifield.

**Results:**

RNFL, GCL, and IPL thickness measurements were significantly reduced in all quadrants, whereas INL, OPL, and PRL thicknesses were significantly increased in the nasal quadrants in patients compared to those in controls. Significant correlations between OCT and 10-2 SAP measurements were positive for the RNFL, GCL, and IPL and negative for the INL, OPL, and PRL. OPs and mfERG N1 amplitudes were significantly reduced in the nasal hemiretina of patients. Significant correlations were found between OP and mfERG amplitudes for inner and outer nasal hemiretina OCT measurements, respectively.

**Conclusion:**

Patients with permanent temporal hemianopia from previously treated chiasmal compression demonstrated significant thinning of the RNFL, GCL, IPL, and thickening of the INL, OPL, and PRL associated with reduced OP and mfERG N1 amplitudes, suggesting that axonal injury to the inner retina leads to secondary damage to the outer retina in this condition.

## Introduction

Optical coherence tomography (OCT) has become a valuable tool not only in the workup of patients with retinal diseases but also in the quantification of retinal neural loss in patients with optic pathway diseases. Reduced thickness of the OCT-measured peripapillary retinal nerve fiber layer (RNFL), macular RNFL, and ganglion cell layer (GCL) thickness are important signs in a number of optic neuropathies, including glaucoma (1–3), optic neuritis from multiple sclerosis (MS) or neuromyelitis optica (NMO) (4–6), and hereditary and compressive optic neuropathies (7–13). Therefore, the quantification of peripapillary RNFL and inner macular layers with high-resolution OCT became a common procedure to diagnose and monitor such conditions.

However, more recently, the widespread use of OCT in optic nerve diseases has revealed retinal abnormalities in layers other than the RNFL and the GCL. Increased thickness and microcystic abnormalities in the inner nuclear layer (INL) were first noticed in MS and NMO (14–18) and later in other optic neuropathies (19–21), in chiasmal compression (22), and in optic tract lesion (23). Although the mechanism has not been clearly established, the presence of INL microcysts in areas of GCL loss points to either mechanical stretching of the retina in the area or retrograde trans-synaptic cell degeneration (19, 20, 22). The observation of secondary INL abnormalities in eyes with anterior visual pathway lesions increases the likelihood of finding abnormalities in the outer retinal layers as well. These layers can now be measured separately with spectral domain OCT (SD-OCT) technology.

Outer retinal layer function may be evaluated with full-field electroretinography (ffERG). The main components of regular ERG data reflect cone and rod responses, while the activity of retinal cells with nuclei located in the INL may be assessed by analyzing the oscillatory potentials (OPs), which are thought to arise preferentially from amacrine cell activity and its interaction with bipolar and ganglion cells (24–29). However, ffERG is a global retinal response and therefore not ideal for evaluating possible localized consequences of retinal axonal loss in optic nerve diseases. Multifocal electroretinography (mfERG), on the other hand, reflects a cone-driven response, predominantly generated by bipolar cell activity from different areas in the central retina (30). In its regular protocol, mfERG is a standard test for the evaluation of outer retinal layer function, but it has been suggested that nonstandard modes of mfERG stimulation and analysis can enhance the contributions of amacrine and bipolar cells in the INL (25). In that regard, modified slow-flash mfERG (SF-mfERG) successfully enhanced OPs, believed to represent intermediate retinal layer activity, in different regions of the macula (31). Previous studies have evaluated inner and intermediate retinal layer function using multifocal OPs (mfOPs) in normal aged persons (30) and patients with retinal diseases (24, 32–36) and glaucoma (37). However, no previous study has specifically addressed the mfERG response in patients with chiasmal disease.

While the inner retinal layers are known to be affected in optic nerve diseases, it remains unclear whether the structures of the outer retinal layer, including the photoreceptor layer (PRL), are also involved. Some histological (38, 39) and electroretinographic (40) studies in glaucoma suggest that the PRL is affected in the disease, but other studies have failed to confirm that (41, 42). In MS patients, Saidha et al. (43) found indications of primary involvement of the outer retinal layer in a subset of the disease, and recent studies indicate that outer retinal changes may be secondary to optic neuritis in that condition (44, 45). However, the effect of primary anterior visual pathway disease on the outer retina is still not well understood. The subject is important, not only from the pathophysiological point of view but also because it helps avoid diagnostic confusion between optic nerve and retinal diseases. Patients with band atrophy (BA) of the optic nerve (46–48) resulting from middle chiasmal compression provide a good model for the investigation of retinal abnormalities secondary to anterior visual pathway disease. Typical cases have temporal hemianopia with normal nasal visual field (VF) (48, 49). Because such patients are affected at the chiasmal level, have no primary ocular diseases and present eyes with temporal hemianopia with normal or near-normal nasal hemifield (with a clear distinction between nasal and temporal hemiretinal involvements), they provide valuable information on possible secondary retinal layer involvement (22, 49). However, to our knowledge, no previous study has used OCT or mfERG to evaluate the segmented outer retinal layers of patients with VF loss from chiasmal compression.

Therefore, the purposes of the present study are to compare segmented inner and outer retinal layers using high-resolution SD-OCT and assess their function using SF-mfERG and regular mfERG recordings in eyes with temporal hemianopia and controls, as well as to determine the relationship between such measurements. We also evaluated the relationship between SD-OCT findings and central VF data from standard automated perimetry (SAP, 10-2-testing strategy) in the same set of patients.

## Materials and Methods

### Subjects

Forty-three eyes of 30 patients with temporal hemianopia from previous chiasmal compression and 37 eyes of 19 healthy controls were included in this observational, prospective, cross-sectional study. All patients had received treatment for suprasellar lesions and had stable visual acuity and VF defects for at least 6 months prior to the study. Approval from the Institutional Ethics Committee was obtained, and all subjects filled informed consent. The study designers followed the principles of the Declaration of Helsinki.

To be eligible, patients were required to (i) be older than 18 years; (ii) have radiologic evidence of a previous suprasellar tumor compressing the chiasm, followed by optic pathway decompression at least 6 months prior to study entry; and (iii) have at least one eye with complete or partial temporal hemianopia and a nasal hemifield within normal limits. An abnormal temporal hemifield was defined on 24-2 VF examination as follows: a cluster of at least three non-edge locations worse than a *P* value of 5%, with at least one worse than a *P* level of 1% on the pattern deviation plot of the temporal hemifield; best-corrected visual acuity of 20/30 or better; spherical equivalent of ±4.00 diopters and IOP < 22 mmHg. Patients with a history of intraocular diseases, diabetes, clinical signs of glaucoma, or other optic neuropathies were excluded.

The control group consisted of healthy subjects recruited among hospital personnel. All individuals had no abnormalities in the ophthalmic examination or the 24-2 SAP. Normal VF was defined as a pattern standard deviation within the 95% confidence limits and a Glaucoma Hemifield Test result within normal limits.

### VF Testing

All subjects were submitted to SAP using the Swedish Interactive Thresholding Algorithm (SITA) Standard 24-2 test (Humphrey Field Analyzer, Carl Zeiss Meditec) with a Goldmann III size stimulus on a 31.5-apostilb background for inclusion purposes. Eligible eyes underwent SITA 10-2 testing, and only reliable VFs were included (fixation loss <20%, false-positive rate <15%, and false-negative rate <30%). The primary analysis consisted of averaging VF deviation from normal values on the total deviation plot in the nasal and temporal hemifields and in the superonasal (SN), inferonasal (IN), superotemporal (ST), and inferotemporal (IT) quadrants. Sectoral mean deviation (MD) and mean sensitivity values were calculated. At each test point, sensitivity values were measured in decibel by averaging the values of the total deviation plot for points in each quadrant and hemifield. Also, for the purpose of calculation, the deviation from normal at each test location was converted from decibel to unlogged 1/Lambert (1/L) units using the formula: 1/L = 10^dB/10^.

### OCT Analysis and Segmentation

**Figure 1:**
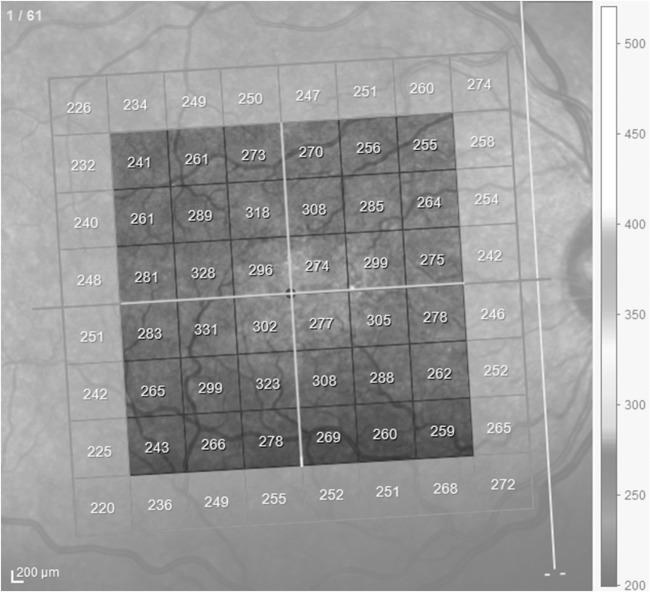
Demarcation of the area in the macula scanned by spectral domain optical coherence tomography. The enhanced squares represent the area used for analysis, divided into four quadrants and two hemifields (white lines). Note the inclination of the scans due to fovea-disk correction. Peripheral squares were excluded from calculations.

Within 2 weeks of VF testing, the subjects were submitted to SD-OCT scanning (Spectralis, Heidelberg Engineering, Heidelberg, Germany) of the macular area following pupil dilation with 1% tropicamide. All participants were examined using the posterior pole protocol of the Spectralis Nsite Axonal Analytics software. A quality index of at least 20 was required for all images. The images were acquired using the automated eye alignment eye-tracking software (TruTrack; Heidelberg Engineering) and corrected with the Fovea-to-Disc Alignment system to obtain macular volumetric retinal scans comprising 61 single vertical lines of 16 frames each, covering a cuboid area of 30° × 25° volume scan (9.2 mm × 7.6 mm) centered on the fovea, to increase the accuracy of horizontal raphe measurements, and to minimize the variation in head orientation. A central 6 mm × 6 mm square of the scanned area was used for analysis (Figure 1). The software scores the quality of the signal strength of the images on a scale from poor (0 dB) to excellent (40 dB). All images were reviewed with regard to subjective and objective quality (50, 51). Criteria for acceptable Spectralis^®^ fundus images included the following: absence of large eye movements (defined as an abrupt shift completely disconnecting a large retinal vessel), consistent signal intensity across the scan, and absence of black bands (caused by blinking) throughout the examination.

**Figure 2:**
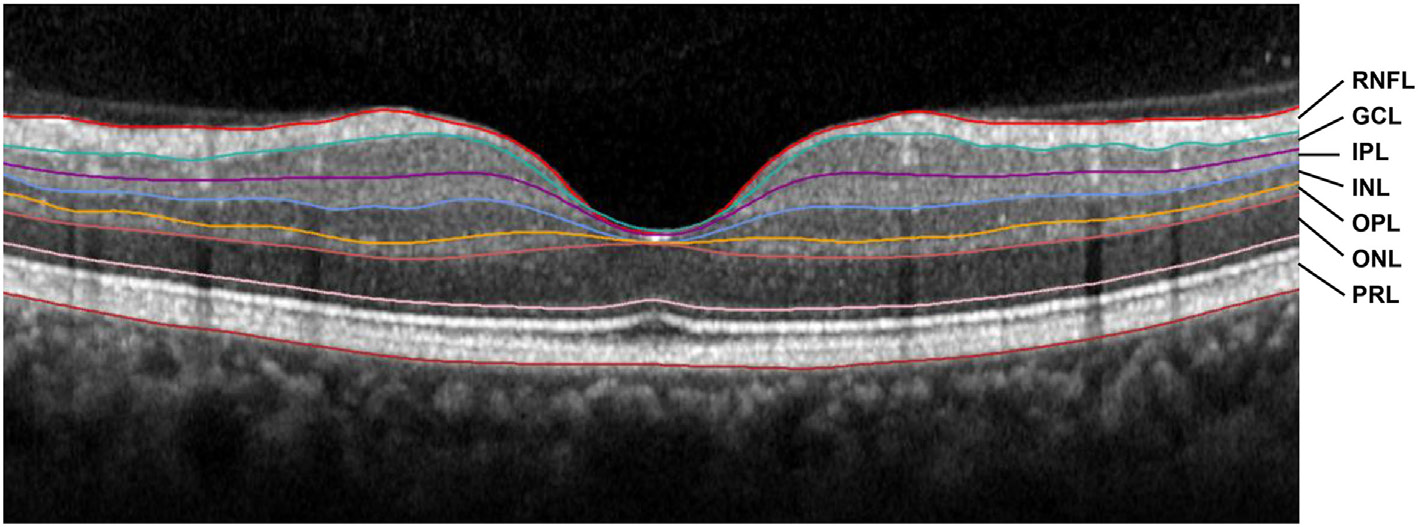
A spectral domain optical coherence tomography scan of the fovea of a normal subject showing the retinal segments measured in this study. The colored lines indicate the boundaries between the layers. RNFL, retinal nerve fiber layer; GCL, ganglion cell layer; IPL, inner plexiform layer; INL, inner nuclear layer; OPL, outer plexiform layer; ONL, outer nuclear layer; PRL, photoreceptor layer.

Seven retinal layers were identified by automatic segmentation performed by the Spectralis^®^ software, with manual correction when needed. After segmentation, we measured the thickness of the following layers between the outer limiting membrane and Bruch’s membrane: the RNFL, the GCL, the inner plexiform layer (IPL), the INL, the outer plexiform layer (OPL), the outer nuclear layer (ONL), and the PRL (52) (Figure 2). The average thickness in macular quadrants and hemifields was calculated for each layer.

### Electroretinography

Multifocal electroretinography was recorded using the RETiscan System (Roland Consult, Wiesbaden, Germany) following the International Society for Clinical Electrophysiology of Vision (ISCEV) guidelines (53). The tests were performed monocularly, after anesthetizing the cornea with one drop of proxymetacaine 0.5%, using ERG-Jet contact lens electrodes (Fabrinal SA, La Chaux de Fonds, Switzerland). Gold-cup reference and surface electrodes were placed on the subject’s forehead and temple, respectively. The pupils were previously dilated with 1% tropicamide, and the subject was light adapted for 10 min before the examination. The stimulus array consisted of 61 hexagons scaled with a 4.0 distortion and eccentricity factor randomly displayed on a CRT monitor at a distance of 26 cm and directed at the central 30° of the retina. The luminance of the stimuli was modulated between black (0 cd/m^2^) and white (200 cd/m^2^), according to a modified pseudorandom m-sequence. The recordings were amplified and automatically bandpass filtered (filter range 10–100 Hz). The mfERG N1 and P1 amplitudes were measured according to ISCEV guidelines (53).

Multifocal OP responses were acquired by SF-mfERG stimulation, with the same 61-hexagon array and the same display used for mfERG recordings. Each hexagon was temporally modulated between black (0 cd/m^2^) and white (200 cd/m^2^) in a pseudorandom binary m-sequence comprising 2^13^-1 steps. The display luminance around the hexagons was approximately 100 cd/m^2^. For this acquisition protocol, the sequence was slowed by inserting three dark frames making each step four frames long (53.3 ms). Each recording session took approximately 13 min per eye, with 98 s per stimulation cycle. Retinal signals were bandpass filtered between 100 and 300 Hz and amplified (range, 100 µV) (54).

**Figure 3:**
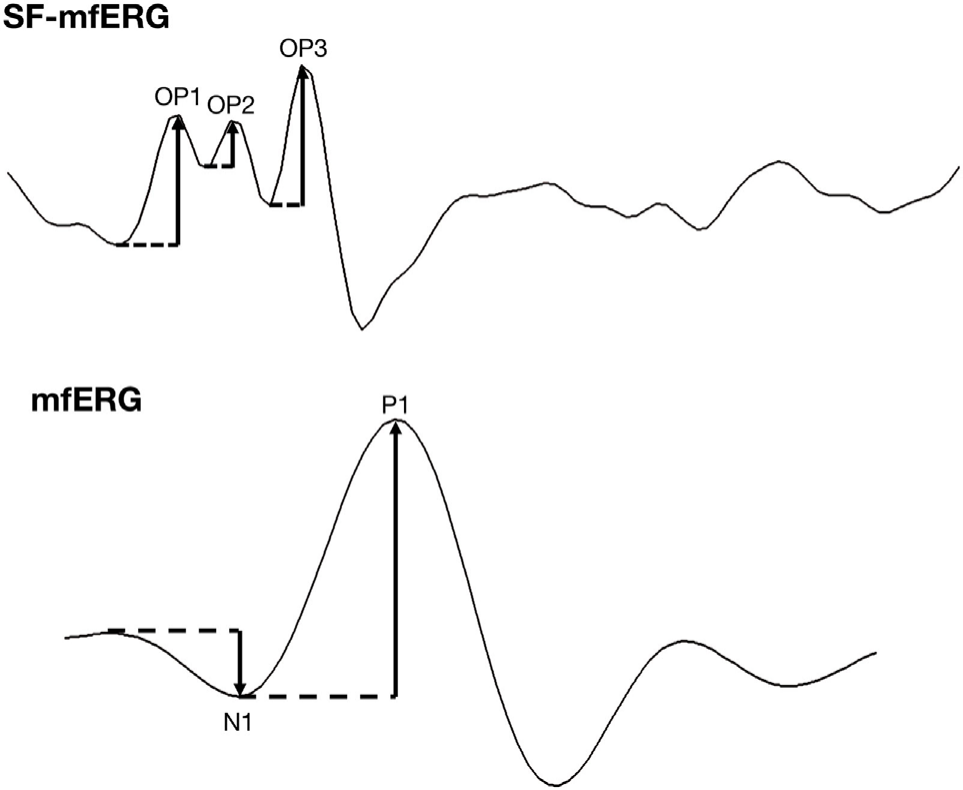
Spatially summed waveforms of regular and slow-flash mfERG of a 34-year-old healthy control. OP amplitude (upper graph) is the difference in nanovolts between a peak and the preceding trough. The final OP amplitude is the sum of the three amplitudes. In the mfERG waveform (lower graph), the N1 amplitude is the difference in nanovolts between the baseline and the first trough; the P1 amplitude is the difference in nanovolts between the first trough and the first peak.

**Figure 4:**
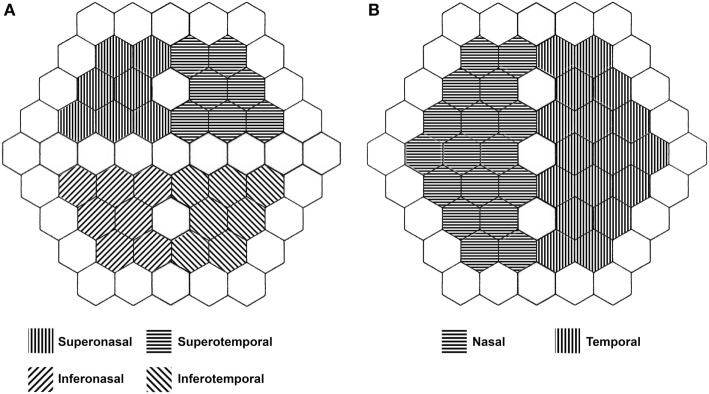
Representation of the 61-hexagon array of retinal stimulation used in both regular and slow-flash mfERG. Note the exclusion of peripheral and midline hexagons from the analysis. **(A)** Quadrantic analysis. **(B)** Hemiretinal analysis.

The subjects were asked to fix their gaze on the red cross at the center of the stimulus screen. Recordings with artifacts from head movements, blinking or contact lens uncoupling were discarded and repeated. The amplitudes of the three major waveforms (P1, P2, and P3) of the SF-mfERG recordings were measured and summed to determine the total OP amplitude (Figure 3). The responses were measured using scaled density regional averages in nV/deg^2^ to reflect the correct angular size for each hexagon stimulating the retina. Disregarding the outermost line of stimulus, SF-mfERG Ops, and conventional mfERG N1 and P1 responses were evaluated for a customized quadrantic pattern (excluding the hexagons in the central vertical and horizontal lines) and for the nasal and temporal hemiretinas (excluding the hexagons in the central vertical line) (Figure 4).

### Statistical Analysis

The statistical analysis of the computed data was performed with IBM SPSS Statistics v. 23.0. The Kolmogorov–Smirnov test was used to confirm the normality assumption. BA patients and normal controls were compared with regard to OCT quadrantic and hemiretinal macular thickness, mean OP amplitudes, and mfERG amplitudes using generalized estimating equations. Receiver operating characteristic (52) curves were used to assess the discrimination ability of each parameter. Correlations between either SD-OCT or electrophysiology measurements and mean SAP sensitivity were verified with either Spearman’s ranked correlation coefficients (ρ) for SAP values in decibels (40) or Pearson’s correlation coefficients (*r*) for SAP values in 1/L units. The level of statistical significance was set at 5% (*P* < 0.05).

## Results

A total of 43 eyes with BA and 37 healthy eyes were studied. All patients had pituitary adenoma. The mean age ± SD was 53.7 ± 13.3 years for the patients and 49.7 ± 12.9 years for the controls (*P* = 0.182, Student’s *t*-test). One subject from the control group had one eye excluded due to a macular scar. On 24-2 SAP testing, 22 eyes presented complete temporal hemianopia, 11 had a defect affecting approximately one quadrant, and 10 had a defect involving less than one quadrant. The mean ± SD of 10-2 SAP MD was −10.38 ± 6.8 dB in the BA group and −1.62 ± 1.2 dB in the control group. In BA eyes, the temporal and nasal mean defect was −18.0 ± 1.2 and −2.6 ± 1.1, respectively. The corresponding values for normal eyes were −1.2 ± 1.0 and −1.1 ± 0.9. The mean defect of the VF quadrants on the 10-2 test was −17.9 ± 11.3 (ST), −2.2 ± 3.5 (SN), −18.1 ± 13.1 (IT), and −2.9 ± 3.3 (IN). The corresponding values for controls were −1.2 ± 1.2, −1.1 ± 1.1, −1.1 ± 0.9, and −1.2 ± 0.8. All VF sensitivity parameters in both hemifields were significantly smaller in BA patients than in controls.

**Figure 5:**
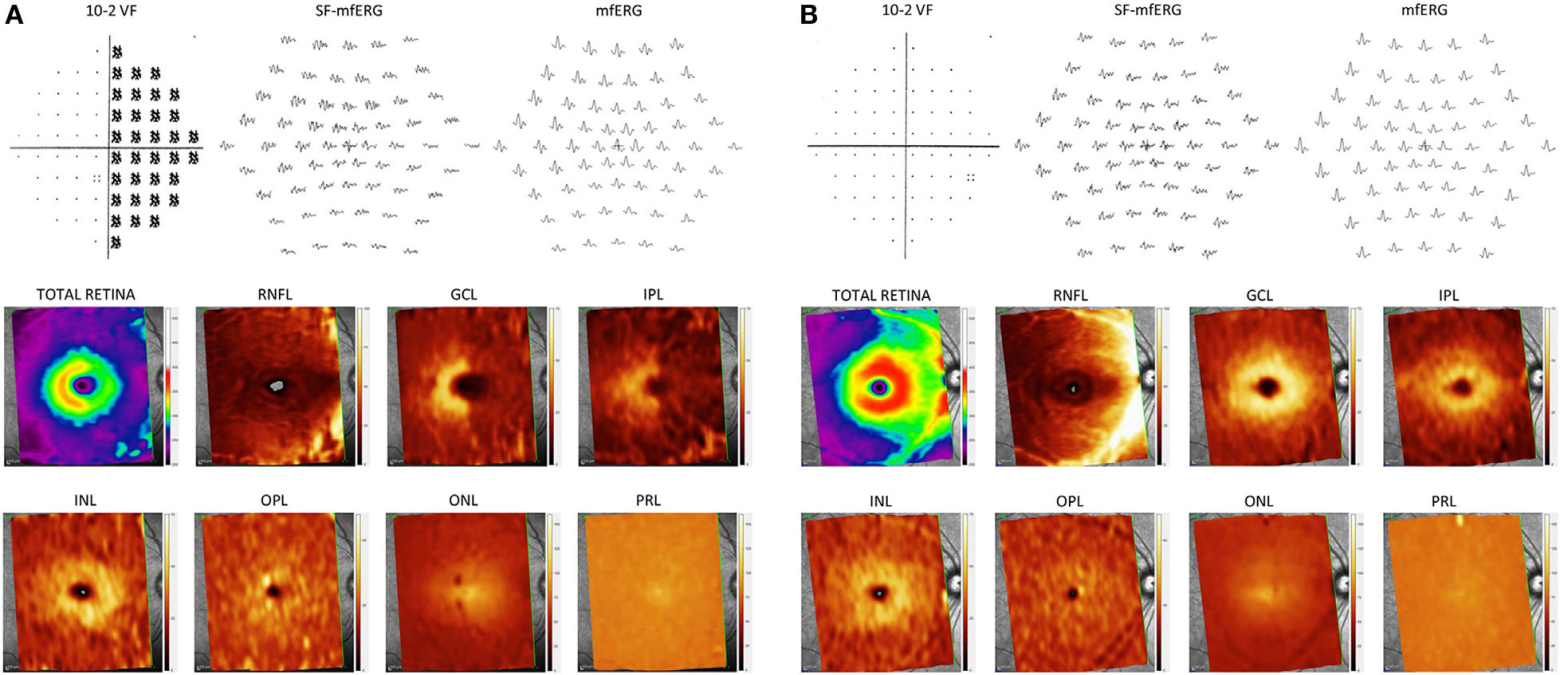
10-2 visual field pattern deviation plot, local responses on regular and slow-flash mfERG and pseudocolor thickness maps generated from the posterior pole OCT scan, based on the thickness of the layers evaluated. **(A)** Patient with band atrophy and temporal hemianopia. Note the amplitude reduction in the nasal sectors on slow-flash mfERG and concomitant increase in outer layer thickness. **(B)** Normal control.

The macular thickness parameters for each retinal layer and each quadrant are presented in Table 1, and the pseudocolor thickness maps of each segmented layer from a typical BA patient and a normal control are shown in Figure 5, with their respective 10-VF total deviation plot and electrophysiological waveforms. RNFL, GCL, and IPL thickness measurements in all four macular quadrants were significantly smaller in eyes with BA than in control eyes. The largest differences were found in the nasal quadrants. These measurements also had the best area under the receiver operating characteristic curve for distinguishing eyes with BA from controls. On the other hand, in the SN and IN quadrants, INL, OPL, and PRL thickness was significantly greater in BA eyes than in control eyes, while no significant difference was found for the temporal quadrants. Moreover, no significant difference was found for ONL measurements, regardless of the quadrant (Table 1).

**Table 1:** Mean values (±SD) of the segmented macular layers thicknesses (in micrometers) by quadrants with AUC.

Parameter	Band atrophy	Controls	*P* value^a^	AUC
**Superonasal quadrant**				
RNFL	32.2 ± 9.5	50.5 ± 8.0	**<0.001**	0.933
GCL	23.5 ± 3.8	34.5 ± 2.3	**<0.001**	0.982
IPL	19.8 ± 2.9	27.6 ± 1.7	**<0.001**	0.977
INL	34.8 ± 3.2	32.4 ± 5.2	**0.015**	0.778
OPL	27.9 ± 3.3	26.4 ± 2.0	**0.009**	0.624
ONL	60.5 ± 5.4	59.3 ± 8.7	0.528	0.494
PRL	81.4 ± 3.0	79.5 ± 2.3	**0.007**	0.692
**Inferonasal quadrant**				
RNFL	38.8 ± 12.2	57.8 ± 7.6	**<0.001**	0.897
GCL	22.6 ± 3.4	33.7 ± 2.3	**<0.001**	0.988
IPL	18.8 ± 2.5	26.7 ± 1.9	**<0.001**	0.987
INL	34.3 ± 2.9	31.1 ± 2.2	**<0.001**	0.809
OPL	27.4 ± 3.3	26.9 ± 2.0	**0.044**	0.613
ONL	58.2 ± 11.2	53.6 ± 8.0	0.086	0.577
PRL	79.7 ± 2.9	78.2 ± 2.3	**0.031**	0.654
**Superotemporal quadrant**				
RNFL	18.8 ± 2.6	20.2 ± 2.2	**0.046**	0.654
GCL	26.6 ± 4.6	33.5 ± 4.6	**<0.001**	0.896
IPL	24.8 ± 3.1	28.5 ± 2.2	**<0.001**	0.837
INL	31.2 ± 2.4	30.4 ± 1.9	0.181	0.619
OPL	26.1 ± 2.1	25.6 ± 1.4	0.263	0.563
ONL	58.0 ± 4.4	58.6 ± 7.7	0.725	0.404
PRL	79.9 ± 2.9	79.2 ± 2.3	0.349	0.570
**Inferotemporal quadrant**				
RNFL	22.4 ± 4.2	25.6 ± 4.1	**0.002**	0.717
GCL	27.8 ± 4.2	33.3 ± 2.8	**<0.001**	0.870
IPL	24.0 ± 2.9	27.5 ± 2.3	**<0.001**	0.833
INL	30.9 ± 2.2	30.3 ± 2.0	0.286	0.595
OPL	25.7 ± 1.6	25.4 ± 1.5	0.572	0.549
ONL	54.8 ± 8.0	54.3 ± 7.4	0.782	0.423
PRL	79.1 ± 2.6	78.6 ± 2.5	0.481	0.553

*^a^Generalized estimating equations*.

*Significant values are in bold*.

*AUC, area under the receiver operating characteristic curve; RNFL, retinal nerve fiber layer; GCL, ganglion cell layer; IPL, inner plexiform layer; INL, inner nuclear layer; OPL, outer plexiform layer; ONL, outer nuclear layer; PRL, photoreceptor layer*.

**Figure 6:**
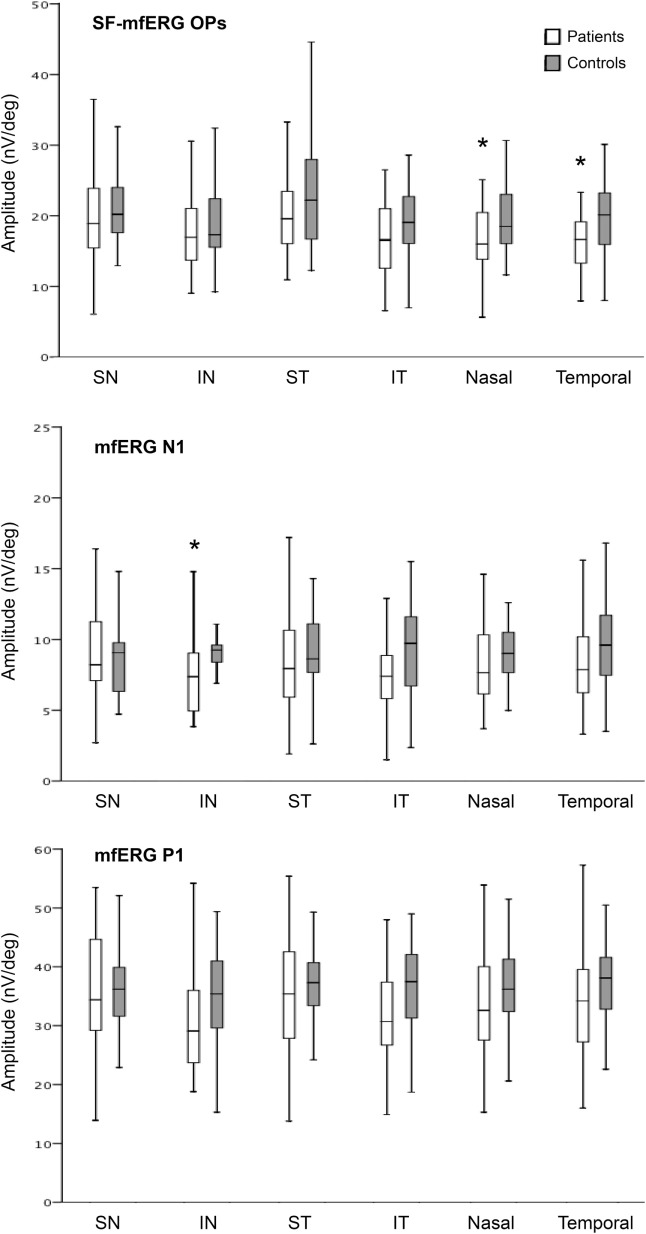
Boxplots of mean values of oscillatory potentials (OP) on slow-flash mfERG and of N1 and P1 amplitudes on regular flash mfERG. Note that the average OP amplitudes of the nasal and temporal hemiretinas were significantly lower in patients with BA than in normal controls. Average N1 amplitude was reduced in the inferonasal quadrant. *p < 0.001 (GEE).

Table 2 and Figure 6 show the results of both regular and SF-mfERG measurements in different quadrants and hemifields of BA eyes and controls. When compared to normals, BA patients had significantly lower mean OP amplitudes in both the nasal and temporal hemiretinas, but no significant difference was found between quadrantic measurements. In BA eyes, the averaged sum of the three positive wavelets was 17.0 ± 5.1 for the nasal hemiretina and 16.8 ± 5.8 for the temporal hemiretina, compared to 19.5 ± 4.7 and 20.1 ± 5.8 in control eyes, respectively. The reduction in N1 and P1 amplitude observed in all quadrants on regular flash mfERG was non-significant except for N1 amplitude in the IN quadrant of BA eyes.

**Table 2:** Mean amplitudes (±SD) of multifocal OPs and multifocal ERG parameters (in nV/deg).

Parameter	Band atrophy	Controls	*P* value^a^	AUC
**Superonasal quadrant**				
mOP	19.6 ± 6.8	21.5 ± 5.4	0.203	0.599
mfERG–N1 amplitude	9.1 ± 3.9	9.0 ± 3.4	0.901	0.522
mfERG–P1 amplitude	36.0 ± 10.7	35.8 ± 6.9	0.951	0.503
**Inferonasal quadrant**				
mOP	18.0 ± 5.8	19.1 ± 5.5	0.437	0.570
mfERG–N1 amplitude	7.4 ± 3.0	8.8 ± 2.6	**0.045**	0.679
mfERG–P1 amplitude	31.3 ± 9.4	35.0 ± 8.0	0.104	0.645
**Superotemporal quadrant**				
mOP	20.7 ± 6.1	23.2 ± 7.2	0.114	0.605
mfERG–N1 amplitude	8.6 ± 3.9	9.5 ± 3.6	0.363	0.580
mfERG–P1 amplitude	35.3 ± 9.8	37.0 ± 6.3	0.444	0.555
**Inferotemporal quadrant**				
mOP	17.7 ± 6.9	17.8 ± 6.0	0.201	0.613
mfERG–N1 amplitude	7.9 ± 3.1	9.6 ± 3.5	0.063	0.649
mfERG–P1 amplitude	32.0 ± 8.6	36.0 ± 8.0	0.059	0.657
**Nasal hemiretina**				
mOP	17.0 ± 5.1	19.5 ± 4.7	**0.029**	0.651
mfERG–N1 amplitude	8.6 ± 3.5	9.1 ± 2.6	0.506	0.602
mfERG–P1 amplitude	34.4 ± 9.9	36.4 ± 7.4	0.373	0.582
**Temporal hemiretina**				
mOP	16.8 ± 5.0	20.1 ± 5.8	**0.013**	0.679
mfERG–N1 amplitude	8.5 ± 3.3	9.8 ± 3.3	0.136	0.638
mfERG–P1 amplitude	34.5 ± 9.0	37.5 ± 7.2	0.167	0.616

*^a^Generalized estimating equations*.

*Significant values in bold*.

*AUC, area under the receiver operating characteristic curve; OP, oscillatory potentials; ERG, electroretinography*.

Table 3 shows the correlations between the thickness of each retinal layer and mean VF sensitivity (1/L) per quadrant on 10-2 SAP. Significant correlations were found between macular thickness (RNFL, GCL, IPL, INL, and PRL) in the nasal quadrants and VF parameters in the temporal quadrants. Correlations with 10-2 sensitivity were positive for RNFL, GCL, and IPL and negative for INL and PRL measurements. The strongest correlations were between GCL thickness in the IN quadrant and VF sensitivity in the ST quadrant (0.831, *P* < 0.01) and between GCL thickness in the SN quadrant and VF sensitivity in the IT quadrant (0.821, *P* < 0.01).

**Table 3:** Correlation between spectralis OCT segmented macular thickness parameters (in micrometers) and mean 10-2 visual field sensitivity parameters (in 1/Lambert).

OCT parameters	10-2 Visual field					
	SN	IN	ST	IT	Nasal	Temporal
**RNFL**						
Superonasal quadrant	0.25	**0.32**	**0.68**	**0.72**	**0.46**	**0.72**
Inferonasal quadrant	**0.30**	0.26	**0.70**	**0.72**	**0.51**	**0.74**
Superotemporal quadrant	0.23	0.18	**0.30**	**0.36**	**0.42**	**0.38**
Inferotemporal quadrant	0.27	0.16	**0.44**	**0.45**	**0.47**	**0.49**
**GCL**						
Superonasal quadrant	**0.29**	**0.42**	**0.83**	**0.82**	**0.42**	**0.83**
Inferonasal quadrant	0.26	**0.40**	**0.83**	**0.80**	**0.37**	**0.81**
Superotemporal quadrant	**0.32**	**0.34**	**0.61**	**0.64**	**0.52**	**0.65**
Inferotemporal quadrant	**0.44**	**0.36**	**0.71**	**0.71**	**0.60**	**0.74**
**IPL**						
Superonasal quadrant	0.22	**0.38**	**0.81**	**0.77**	**0.30**	**0.79**
Inferonasal quadrant	0.16	**0.38**	**0.79**	**0.74**	0.26	**0.75**
Superotemporal quadrant	**0.35**	**0.33**	**0.65**	**0.65**	**0.53**	**0.69**
Inferotemporal quadrant	**0.35**	**0.30**	**0.67**	**0.66**	**0.48**	**0.70**
**INL**						
Superonasal quadrant	−0.11	−0.11	−0.26	−0.28	−0.21	−0.25
Inferonasal quadrant	−0.18	−0.18	−**0.49**	−**0.48**	−**0.31**	−**0.46**
Superotemporal quadrant	0.05	−0.06	−0.12	−0.09	−0.05	−0.06
Inferotemporal quadrant	0.04	0.01	−0.08	−0.04	−0.05	−0.02
**OPL**						
Superonasal quadrant	−0.01	0.02	−0.18	−0.22	−0.07	−0.19
Inferonasal quadrant	−0.00	0.07	−0.07	−0.12	−0.03	−0.11
Superotemporal quadrant	0.01	0.10	−0.17	−0.17	−0.07	−0.11
Inferotemporal quadrant	0.00	0.12	−0.11	−0.09	−0.05	−0.08
**ONL**						
Superonasal quadrant	0.01	−0.02	−0.13	−0.11	−0.06	−0.15
Inferonasal quadrant	−0.08	−0.07	−0.14	−0.12	−0.14	−0.15
Superotemporal quadrant	0.04	0.06	0.07	0.06	0.02	0.03
Inferotemporal quadrant	0.02	0.06	−0.02	0.06	0.01	0.02
**PRL**						
Superonasal quadrant	−0.12	−0.14	−**0.29**	−**0.29**	−0.09	−**0.29**
Inferonasal quadrant	−0.08	−0.11	−0.27	−**0.27**	−0.07	−**0.31**
Superotemporal quadrant	−0.13	−0.14	−0.18	−0.19	−0.12	−0.19
Inferotemporal quadrant	−0.12	−0.14	−0.15	−0.16	−0.08	−0.16

*Pearson’s correlation coefficient. Underline type = P < 0.05; bold type = P < 0.01*.

*OCT, optical coherence tomography; SN, superonasal quadrant; IN, inferonasal quadrant; ST, superotemporal quadrant; IT, inferotemporal quadrant; RNFL, retinal nerve fiber layer; GCL, ganglion cell layer; IPL, inner plexiform layer; INL, inner nuclear layer; OPL, outer plexiform layer; ONL, outer nuclear layer; PRL, photoreceptor layer*.

The results of the Spearman correlation analysis between segmented macular thickness and electrophysiological measurements are shown in Table 4. A positive significant correlation was found only between OP amplitude and INL thickness in the SN quadrant.

**Table 4:** Correlation between spectralis OCT segmented macular thickness parameters (in micrometers) in quadrants and hemiretinas and their respective OPs on slow-flash mfERG and amplitudes (nV/deg) on regular mfERG.

Electrophysiology parameters	OCT parameters					
	SN	IN	ST	IT	Nasal	Temporal
**RNFL**						
OP	0.213	0.201	0.124	0.144	0.28	0.19
mfERG–N1	0.06	0.20	−0.05	0.11	0.12	0.09
mfERG–P1	0.09	0.21	−0.11	0.08	0.15	0.02
**GCL**						
OP	0.17	0.22	0.25	0.28	**0.31**	**0.33**
mfERG–N1	0.04	**0.34**	0.14	0.17	0.18	0.17
mfERG–P1	0.13	**0.29**	0.15	0.19	0.22	0.17
**IPL**						
OP	0.10	0.17	0.17	0.19	0.26	0.23
mfERG–N1	0.38	**0.37**	0.08	0.13	0.18	0.12
mfERG–P1	0.14	**0.30**	0.09	0.12	0.22	0.12
**INL**						
OP	0.23	0.03	0.10	0.12	−0.19	0.04
mfERG–N1	−0.05	0.02	0.09	0.10	−0.01	0.08
mfERG–P1	0.10	0.04	0.11	0.16	0.05	0.16
**OPL**						
OP	−0.03	0.10	−0.09	0.00	0.09	0.02
mfERG–N1	−0.00	−0.02	0.06	0.07	0.01	0.05
mfERG–P1	0.09	0.14	0.09	0.12	0.09	0.08
**ONL**						
OP	−0.11	−0.07	0.01	−0.03	−0.15	0.03
mfERG–N1	0.14	−0.08	0.19	0.23	−0.03	0.23
mfERG–P1	0.13	−0.01	0.24	0.11	−0.06	0.18
**PRL**						
OP	−0.14	−0.10	0.01	−0.01	−0.18	−0.03
mfERG–N1	−0.06	−0.14	−0.06	−0.15	−0.12	−0.12
mfERG–P1	0.10	−0.21	−0.01	−0.10	0.03	−0.07

*Spearman’s correlation coefficient. Underline type = P < 0.05; bold type = P < 0.01*.

*OCT, optical coherence tomography; SN, superonasal quadrant; IN, inferonasal quadrant; ST, superotemporal quadrant; IT, inferotemporal quadrant; OP, oscillatory potential; RNFL, retinal nerve fiber layer; GCL, ganglion cell layer; IPL, inner plexiform layer; INL, inner nuclear layer; OPL, outer plexiform layer; ONL, outer nuclear layer; PRL, photoreceptor layer; mfERG, multifocal electroretinography*.

## Discussion

Our findings confirm that in patients with sequel temporal hemianopia due to chiasmal compression, RNFL, GCL, and IPL thickness is reduced not only in the nasal quadrants but also, to a certain extent, in the temporal quadrants of the macular area (Table 1). Despite the rationale that our patients with temporal hemianopia and nasal VF within normal limits should have normal uncrossed nerve fibers and normal retinal structures temporal to the macula, we found reduced inner retinal layers in both the nasal and the temporal hemiretina, matching previous studies in similar sets of patients (7, 11, 13, 55).

Presumably, damage to the retinal ganglion cell (RGC) and retinal nerve fibers originating in the temporal hemiretina occurred before chiasmal decompression, and although the nasal VF was labeled as being within normal limits at the time of the study, some subclinical damage already existed in the temporal retina. Despite this limitation, our model is interesting in that it allows to compare clearly (often severely) affected nasal hemiretinas to relatively preserved temporal hemiretinas. Therefore, the model seems valid for the main purpose of the current study that was to investigate structural and functional changes in retinal layers other than the RNFL and the GCL in patients with temporal hemianopia from previous chiasmal compression.

In a previous study, increased INL thickness (measured together with the OPL) in segmented SD-OCT measurements in patients with chiasmal compression was limited to the nasal hemiretina (22). This observation was confirmed in the present study by the finding of increased thickness in both the INL and OPL (measured separately), supporting the notion that primary damage to the RNFL and GCL caused by axonal degeneration from chiasmal compression is associated with secondary structural abnormalities in the INL (Table 1). We also observed functional abnormalities at the level of the INL, as indicated by the significantly lower mean mfOP amplitude in the affected nasal hemiretina (Table 2). To enhance the OP components from specific areas of the macula, we used SF-mfERG (inserting periods of darkness between the stimuli). Previous pharmacological (28) and clinical (24, 36, 56, 57) studies suggest that mfOPs are a sign of INL cell activity (especially from amacrine and bipolar cells), with some influence of rod and cone interaction (31). Decreased mfOP amplitude has been documented in other conditions known to affect the inner retina, such as diabetic retinopathy (35), progressive myopia (32), and glaucoma (37). The current study is the first to document reduced mfOP values on SF-mfERG along with abnormal OCT structural measurements in the INL of BA patients (Tables 1 and 2). In a previous study, increased INL thickness in the nasal retina of BA patients was associated with a high incidence of microcystic INL abnormalities (22), a phenomenon observed in studies on other optic neuropathies (17, 20, 58–60). In such studies, retinal microcysts were more prominent in the parafoveal area, corresponding to the area with the largest concentration of both Müller and ganglion cells (61). Researchers (20, 62) have suggested that vitreous traction could be associated with the occurrence of INL microcysts, but we find it an unlikely pathogenic factor, since in a previous study, the microcysts were restricted to the nasal hemiretina of eyes with temporal hemianopia and BA of the optic nerve (22), making it unlikely that the vitreous would draw only the hemiretina with severe GCL loss. In view of the importance of Müller cells for the maintenance of the retinal framework, we believe that RGC atrophy may have a tractional effect on Müller cells, resulting in increased thickness in areas with RGC damage. Van Buren (63) found histological evidence of retrograde trans-synaptic degeneration in the INL of primates and humans after optic nerve damage, with cavitary degeneration in the inner half of INL, suggesting damage of the amacrine and Müller cells. However, according to Gills and Wadsworth (64), this pattern of degeneration was more likely caused by bipolar cell loss since the amount of cell loss was incompatible to amacrine or Müller cell damage alone. These findings from the 1960s resemble the microcystic abnormalities observed on OCT today (22). The present findings of INL enlargement on OCT and reduced OP on mfERG support the hypothesis that optic pathway damage leads to abnormalities in the RGC and possibly also in the retinal cells of the INL. In fact, our study points to a combination of mechanical stretching of the retinal structures and secondary degeneration of the bipolar, amacrine, and Müller cells in the INL.

To investigate other potential secondary abnormalities in the retina, we evaluated the outer retinal structures by analyzing segmented OCT-measured outer retinal layers and mfERG data. An isolated reduction in N1 amplitude was found in the macular IN quadrant, and PRL thickness was greater in the SN and IN quadrants. However, the P1 amplitude did not differ significantly between BA and normal subjects regardless of the quadrant. To date, no study has investigated the presence of outer retinal abnormalities in BA patients, but evidence of photoreceptor damage in glaucoma and other optic neuropathies by authors using adaptive optics have been reported, with good correlation between areas of ganglion cell loss and lower cone density (65). Ultrahigh-resolution OCT (66) and multiphoton imaging of stained tissue (67) have also revealed PRL abnormalities in glaucoma patients. Despite the differences between conventional ERG and mfERG, the mfERG N1 wave response is thought to correspond to a multifocal form of the full-field ERG a-wave component, indicating photoreceptor activity in the outer retina (68). Recent OCT studies have described changes in outer retinal structures following optic neuritis, with thickening of the outer retinal layers, especially in severe cases (44, 45). Nonetheless, no previous study has evaluated the outer retina in other optic neuropathies, and the actual mechanism responsible for these abnormalities remains unclear.

In the current study, the PRL thickness increased only in the nasal hemiretina of BA patients, along with an isolated N1 amplitude reduction in the IN quadrant, suggesting some mechanical stretching of the outer retinal layers, with some cell injury. Since the only abnormality of mfERG was the reduction of N1 amplitude in the IN retinal quadrant, it is not possible to affirm that there is also trans-synaptic degeneration of the photoreceptors in our set of patients. On the other hand, since the IN retinal quadrant is the most extensively damaged sector in patients with chiasmal compression (which tends to affect the ST VF quadrant), it is possible to infer from the abnormal N1 amplitude observed in the corresponding IN quadrant that secondary photoreceptor degeneration does in fact occur but only when a certain level of retinal damage is reached, therefore affecting only the most heavily damaged retinal quadrant. Further studies are necessary to better understand these findings.

We also evaluated the association between OCT/mfERG measurements and VF data in our patients with chiasmal compression, using the 10-2 SAP testing strategy. Previous studies evaluating structure-function relationships in such patients have used OCT measurements plotted against data obtained with the 24-2 strategy or the 30-2 strategy (7, 8, 11, 22, 69, 70). Although these strategies may seem adequate for investigating structure-function relationships with OCT-derived peripapillary RNFL thickness measurements, they provide little information to correlate with macular thickness measurements, which are usually based on a 6 mm × 6 mm central area around the fovea. On the other hand, the 10-2 testing strategy evaluates 68 test points in the central 10° providing much more information within the macular area (61). While significant attention has been given to central VF testing in glaucoma (71, 72), no previous study has assessed the correlation between 10-2 SAP testing and SD-OCT or ERG data in chiasmal compressive lesions. In the current study, the correlation between changes in OCT-measured thickness and 10-2 SAP sensitivity was positive for the inner layers with reduced thickness and negative for layers with increased thickness (INL and PRL). The strongest correlation was between SAP and GCL, matching data from previous 24-2 VF studies (22). The coefficients were slightly greater in the present study (up to 0.83 for the best-performing measurements) than in our previous study, using the 24-2 test in a similar set of patients (22), suggesting that more central VF data would be useful in the evaluation of patients with chiasmal compression, but the difference was not large enough to allow for inferences. However, it is important to stress that our study was conducted on patients with permanent temporal hemianopia secondary to chiasmal compression selected based on the presence of abnormalities on 24-2 VF testing. Thus, it would be interesting to compare the 10-2 and the 24-2 testing strategy with regard to early detection in patients with suspected chiasmal compression. As for OCT and mfERG, significant correlations were found between mfOPs and OCT-measured RNFL, RGC, and INL thickness in the nasal hemiretina. No significant correlation was found between OCT and regular mfERG-measured N1 and P1 amplitudes except for a correlation with ONL thickness in the temporal retinal quadrants (Table 4). We believe that the abnormalities in the outer retinal layers were too subtle to yield a clinically significant correlation.

In conclusion, our study shows that axonal damage from BA of the optic disk leads to significant thinning of the RNFL, GCL, IPL, and thickening of the INL, OPL, and PRL in relation to healthy eyes, and such eyes can be distinguished from normal eyes by quadrantic analysis of the retinal layers. We also observed changes in OP and mfERG values in the nasal macula of patients with chiasmal compression, indicating functional injury to both the inner and the outer retina. Our results point to the combination of a trans-synaptic mechanism of degeneration and mechanical stretching throughout the retina, but the current data are insufficient to confirm this conclusion, and further studies are necessary to verify it.

## Author Contributions

RA: conception, design, collection of data, analyses of data, writing of the manuscript, and revising the manuscript. MO: conception, design, collection of data, analyses of data, and revising the manuscript. LZ: providing equipment for the study, conception of the study, and revising the manuscript. LC: conception, design, and revising the manuscript. RP: conception, writing the manuscript, and revising the manuscript. MM: providing equipment for the study, conception, design, collection of data, analyses of data, writing of the manuscript, and revising the manuscript.

## Conflict of Interest Statement

The authors declare that the research was conducted in the absence of any commercial or financial relationships that could be construed as a potential conflict of interest.
